# Comparative evaluation of isolation techniques and characterization of red pulp macrophages from pig splenocytes

**DOI:** 10.3389/fimmu.2025.1617203

**Published:** 2025-09-01

**Authors:** Phu Chi Vu, Nhat Minh Dang, Jonghyeok Jung, Min Hyuk Kim, Min Guk Lee, Joohyun Shim, Thi Xoan Hoang, Jae Young Kim

**Affiliations:** ^1^ Department of Life Science, Gachon University, Seongnam, Kyeonggi-Do, Republic of Korea; ^2^ Department of Transgenic Animal Research, Optipharm Inc., Cheongju, Republic of Korea; ^3^ Nguyen Tat Thanh (NTT) Hi-tech Institute, Nguyen Tat Thanh University, Ho Chi Minh, Vietnam

**Keywords:** pig spleen, red pulp macrophages, autofluorescence, CD163, RBC clearance, iron retention

## Abstract

**Introduction:**

Red pulp macrophages (RPMs) play a central role in iron recycling and immune regulation within the spleen, yet optimized methods for the isolation and characterization of pig RPM remain limited.

**Methods:**

We compared two approaches for isolating RPMs from pig splenocytes: CD163 antibody- based sorting and magnetic-activated cell sorting (MACS), which leverages the natural iron content and autofluorescence of RPMs. Isolated cells were evaluated by flow cytometry for marker expression, and functional assays were performed to assess phagocytic activity and gene expression related to iron metabolism.

**Results:**

Flow cytometry identified an autofluorescent population, a hallmark of RMPs, within the pig splenocytes. CD163-based method enriched RPMs to 71.8% autofluorescent cells, while the MACS- based approach achieved a higher yield of 81% autofluorescent cells without using antibodies, demonstrating greater cost-effectiveness and efficiency. Marker analysis revealed high expression of CD16 and CD163, moderate expression of CD11b, and low or undetectable levels of CD14, CD32, and CD169. Functionally, isolated RPMs demonstrated robust phagocytosis of senescent red blood cells and upregulation of genes involved in heme and iron metabolism.

**Discussion:**

These findings establish an optimized, antibody-free protocol for efficient isolation of pig RPMs. The approach provides a reliable platform for studying splenic macrophage biology, iron homeostasis, and immunological research and splenic function studies.

## 1 Introduction

Spleen plays a crucial role in the immune system, particularly in the filtration of blood and the removal of aged or damaged red blood cells (RBCs) (1). One of its primary functions is to clear senescent RBCs, preventing the accumulation of dysfunctional cells that could impair oxygen transport. In this context, red pulp macrophages (RPMs) within the spleen are key players.

RPMs are highly efficient in phagocytosing and degrading aged RBCs. In addition to RBC clearance, they are central to iron homeostasis, recycling iron from heme to support erythropoiesis (2, 3). Dysregulation in this recycling process can lead to anemia or iron overload (4, 5). Thus, RPMs contribute critically to both immune defense and systemic iron metabolism (6; 7, 8).

RBC clearance is initiated by opsonization with naturally occurring antibodies, allowing Fcγ receptor-mediated phagocytosis by RPMs (8, 9).

Previous studies have reported various markers and isolation strategies for RPMs across species. In pigs, RPMs have been defined as CD163^+^ cells by immunofluorescence (10) and further isolated using magnetic CD163-beads, revealing their immune profile (11). In humans, RPMs are typically identified as autofluorescent CD163^+^CD16^+^ cells via FACS (3, 12). In mice, RPMs have been isolated based on their magnetic properties stemming from iron accumulation, with autofluorescence used as a hallmark for identification (13).

Given the anatomical and immunological similarities between pigs and humans (14, 15), optimizing methods to isolate and characterize pig RPMs is valuable for translational research.

In this study, we optimized and compared two strategies for isolating pig RPMs: (1) CD163 antibody-based magnetic sorting, and (2) magnetic property-based separation leveraging their iron content, validated by autofluorescence. While autofluorescence served as a confirmation of RPM identity, the actual MACS-based isolation was driven by intrinsic magnetic properties due to iron content, not fluorescence. We evaluated the efficacy of both methods by quantifying CD163 expression and autofluorescent cell proportions, then characterized surface markers and functional properties related to erythrophagocytosis and iron metabolism. This work establishes a robust protocol for pig RPM isolation and expands our understanding of their biological features and relevance for biomedical research.

## 2 Materials and methods

### 2.1 Animal

Splenocytes and peripheral blood mononuclear cells (PBMCs) were isolated from genetically modified triple-knockout (TKO) pigs obtained from Optipharm Co., Ltd. (IACUC approval number: OPTI-IAC-2301). Peripheral blood was collected via jugular venipuncture using K2-EDTA treated vacutainers, and splenic tissues were harvested immediately after euthanasia under approved protocols. General anesthesia was induced by using ketamine (20 mg/kg) and xylazine (2.3 mg/kg) administered intramuscularly, followed by maintenance with isoflurane. Euthanasia was performed via intravenous infection of 1 M potassium chloride (KCl) solution prior to splenectomy.

All procedures were conducted in accordance with the guidelines and ethical standards approved by the Institutional Animal Care and Use Committee (IACUC) of Optipharm.

For each experiment, splenocytes were isolated from an individual pig (n = 3 pigs).

### 2.2 Preparation of splenocytes and experimental design

Splenocytes were obtained by mechanical dissociation of a small piece (approximately 0.2 cm) of the splenic tissue followed by filtration through a 100-µm cell strainer. For each experiment, splenocytes were freshly isolated from a single pig spleen. The total single-cell suspension obtained was evenly divided into two equal parts to ensure a direct comparison of the two RPM isolation methods under identical conditions. One half was used for MACS-based isolation, and the other half was subjected to CD163-based isolation method. The yield and enrichment efficiency were evaluated as the percentage of autofluorescence-positive cells among the total cells.

Peripheral blood mononuclear cells (PBMCs) were isolated by density gradient centrifugation using Ficoll-Paque (Cytiva, Sweden) and used as a negative control.

### 2.3 MACS-based isolation of RPM from pig spleen

To isolate RPMs using their magnetic properties, single-cell suspensions of splenocytes were applied to LS Separation Columns (Miltenyi) and washed three times with 3 mL of MACS buffer (Miltenyi) while on the magnetic stand to remove unbound cells. After detaching the column from the magnet, retained RPMs were eluted using 5 mL of MACS buffer for downstream analysis.

### 2.4 CD163-based isolation of RPM from pig spleen

CD163^+^ macrophages were isolated from pig splenocytes using a MACS column. The splenocytes were incubated with anti-pig CD163 monoclonal antibodies, followed by a secondary incubation with anti-Ig microbeads. The cell mixture was then passed through an LS magnetic column (Miltenyi Biotec), allowing for the selective retention of CD163^+^ cells. The positively selected CD163^+^ macrophages were subsequently eluted, and the purity of the isolated population was determined by flow cytometry.

### 2.5 Assessment of autofluorescent population

Autofluorescence in the splenic cell population was assessed using fluorescence microscopy without any labeled antibodies or fluorescent dyes. Specifically, splenic cells, both pre-isolation and post-isolation, were directly visualized under the fluorescence microscope without prior treatment. Furthermore, autofluorescence was quantified by flow cytometry. The gating strategy for flow cytometry analysis consisted of three sequential steps. First, the main splenocyte population was identified by analyzing forward scatter (FSC-A) and side scatter (SSC-A) parameters, which allowed for the exclusion of debris and small particles. Second, single cells were selected by gating on the diagonal population in a forward scatter height (FSC-H) versus forward scatter area (FSC-A) plot, effectively eliminating doublets and aggregates. Finally, autofluorescent cells were identified by assessing their emission profiles in FL-1 and FL-2 channels. Autofluorescence was quantified by selecting cells that were double-positive for FL-1 and FL-2 channels, indicating intrinsic fluorescence in the absence of external fluorophores.

### 2.6 Immunofluorescent staining

To assess the morphology of RPMs, isolated cells were cultured in DMEM supplemented with 10% FBS, 100 U/mL penicillin (Gibco), and 100 μg/mL streptomycin (Gibco) for 3 days. Cell morphology was observed using an inverted microscope (Olympus, CKX53, Tokyo, Japan). To evaluate CD163 expression on RPMs, positive cells after isolation were incubated with PE-conjugated anti-pig CD163 antibodies for 30 minutes at 37°C. Cells were washed twice with D-PBS, and the images were captured using a fluorescence microscope (Olympus, CKX53, Tokyo, Japan).

### 2.7 Iron detection

For staining of ferric iron, both positive and negative fractions from splenocytes and PBMCs were plated on glass coverslips. The cellular ferric iron was stained using an iron staining kit (Cat. Ab150674, Abcam) according to the manufacturer’s protocol. The images were captured using a fluorescence microscope (Olympus, CKX53, Tokyo, Japan). To evaluate relative iron levels in different fractions, the oxidation of Amplex UltraRed (AUR) with H_2_O_2_ was performed in a 96-well plate (Black, Greiner Bio-One, Courtaboeuf, France). Briefly, after isolation, cells from both negative and positive fractions were lysed using 2% Triton X. The supernatants were collected and used for the AUR assay. In each well, a reaction mixture containing 105 µL of Tris-acetate buffer (100 mM, pH 7.0), 15 µL of cell lysate, 15 µL of 10 mM H_2_O_2_, and 15 µL of 500 µM AUR was added. The plate was then incubated at room temperature for 10 minutes. Following incubation, fluorescence intensity was measured using a microplate reader at an excitation/emission of 530/580 nm (Synergy H1, BioTek, VT, USA).

### 2.8 Flow cytometry

To determine cell surface protein expression, the cells were incubated with fluorescence-conjugated anti-CD14 (Miltenyi), anti-CD16 (BD Biosciences), anti-CD32 (Arigo Biolaboratories), anti-CD163 (Invitrogen), and anti-CD169 (Invitrogen) for 30 minutes at 4°C. After each step, samples were washed three times with DPBS. Finally, samples were diluted in 0.4 mL of DPBS and analyzed using a Cytomics FC500 MLP flow cytometer (Beckman Coulter Inc., Fullerton, CA, USA). The data obtained were analyzed using FlowJo™ Software (Treestar Inc., Ashland, USA).

### 2.9 Phagocytosis assay

Triple-knockout (TKO) pig RBCs were provided by Optipharm Inc. (Cheongju, Korea). Freshly isolated RBCs or RBCs stored at 4°C for 6 months were used to assess phagocytic function. The RBCs were stained with 1 µg/mL calcein AM cell-permeant dye (ThermoFisher Scientific, cat#1430) for 1 hour at room temperature, followed by two washes with DPBS to remove excess dye. The labeled RBCs were then incubated at 50°C for 20 minutes to induce senescence. Following MACS sorting, cells from either negative or positive fractions were co-incubated with the heat-treated RBCs for 1 hour at 37°C to assess phagocytic activity; incubation at 4°C was used as a negative control. After incubation, unengulfed RBCs were removed using an RBC lysis buffer. The cells were then lysed with 1% Triton X solution, and fluorescence intensity was measured using a microplate reader at an excitation/emission wavelength of 488/520 nm.

### 2.10 RNA preparation and quantitative real-time polymerase chain reaction for gene expression

Total RNA was extracted from both negative and positive fractions using the easy-BLUE™ Total RNA Extraction Kit (iNtRON Biotechnology, INC.) according to the manufacturer’s instructions. RNA purity and concentration were measured using a MaestroNano MicroVolume Spectrophotometer (Maestrogen, Las Vegas, NV, USA). Two micrograms of RNA was reverse-transcribed into cDNA using the Hyperscript™ 2X RT Master Mix (Geneall Biotechnology).

qPCR was performed on a Rotor-Gene system (Qiagen) using EzAMP™ FAST One-Step RT-qPCR 2× Master Mix (SYBR; Elpis-Biotech Inc., Daejeon, Republic of Korea) with the following primer sets:

NENF 5′-TATCCCATCGTCGGCTACAC-3′, 5′-TCAACGTCTGCTTTGTTCGG-3′; HMOX-1 5′-AAGATTGCTCAGAAGGCCCT-3’, 5’-TTAGTGTCCTGGGTCAGCAG-3’; FTH1 5’-CATCAACCGCCAGATCAACC-3’, 5’-AGCCCATTCTCCCAGTCATC-3’; GAPDH 5’-ACCCAGAAGACTGTGGATGG -3’, 5’-GTCCTCAGTGTAGCCCAGGA-3’; EEF1A1 5′-CATTGATGCTCCAGGACACA-3′, 5’-CCGTTCTTGGAAATACCTGCTT-3’; HPRT1 5’-AAGGACCCCTCGAAGTGTTG-3’, 5’-CACAAACATGATTCAAGTCCCTG-3’.

To ensure reliable normalization, we evaluated the expression stability of three commonly used housekeeping genes – GAPDH, EEF1A1, and HPRT1 – across our experimental samples. GAPDH was selected based on its stable expression compared to the other candidates, as determined by comparative Ct analysis and gene stability ranking using NormFinder.

qPCR reactions were performed in triplicate for each sample. Melt curve analysis was conducted at the end of each run to confirm application specificity. Relative gene expression levels were calculated using the 2^−ΔΔCt method, with GAPDH used as the endogenous reference gene. All procedures were carried out in accordance with MIQE guidelines (16).

### 2.11 Statistical analysis

The experiments were conducted at least three times, and all data are presented as mean ± standard deviation (SD). Significant differences among groups were analyzed by two-way analysis of variance (ANOVA) followed by Tukey’s *post hoc* test using GraphPad Prism 8.0.2 software (La Jolla, CA, USA). For other statistical analyses, p-values were calculated using Student’s t-test. A p-value of <0.05 was considered statistically significant.

## 3 Results

### 3.1 Comparison of RPM isolation methods

**Figure 1:**
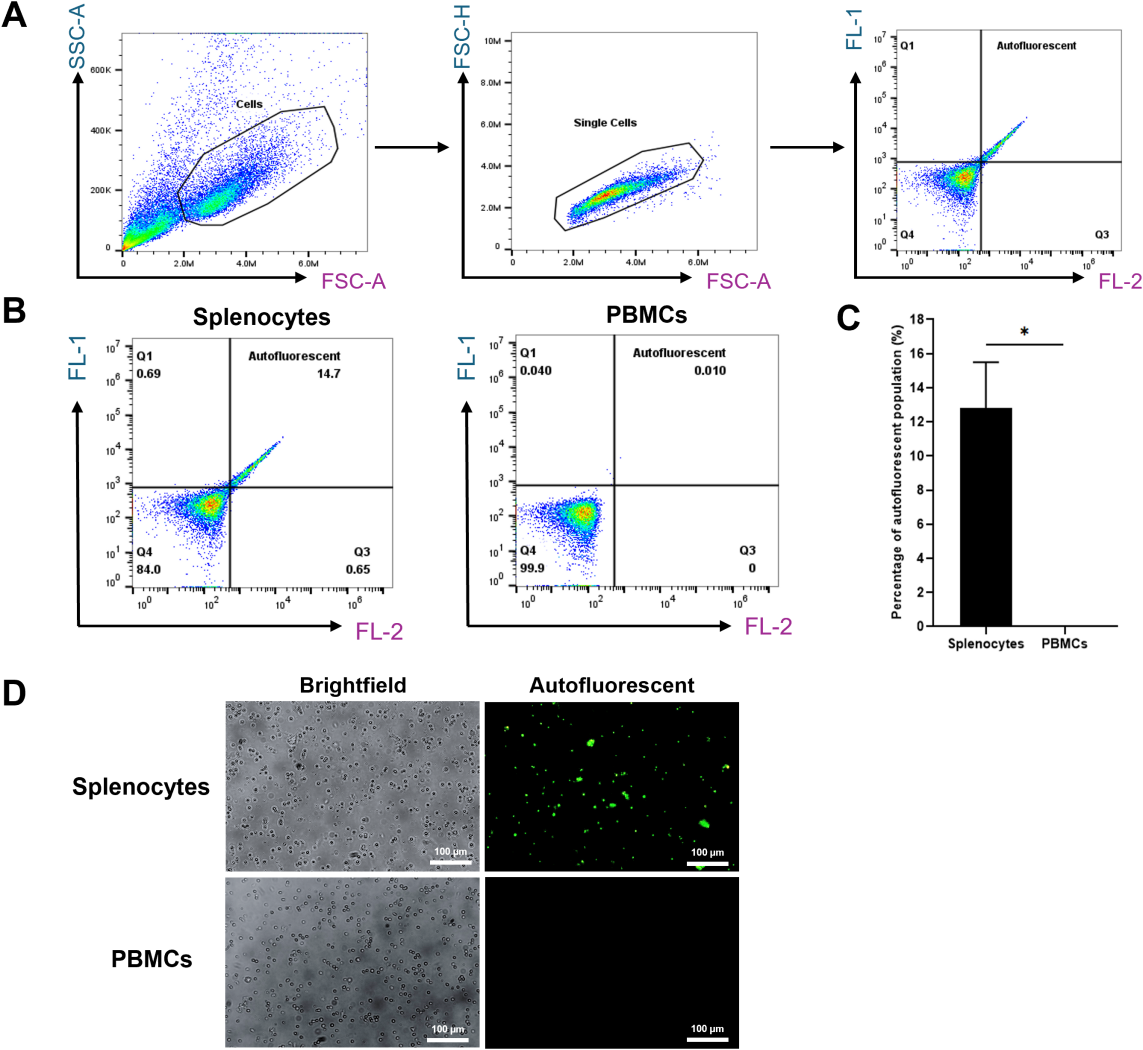
Autofluorescent cell population in splenocytes. **(A)** Gating strategy used to assess the autofluorescent population. **(B)** Representative flow cytometry plots of unstained single-cell suspensions of splenocytes (left) and PBMCs serving as a negative control (right). **(C)** Percentage of autofluorescent populations in splenocytes and PBMCs. **(D)** Fluorescence microscopy images (200X magnification) showing autofluorescent cells in splenocytes, with PBMCs included for comparison. *p < 0.05. n = 3 independent experiments using cells from three individual pigs.

We first analyzed total splenocyte populations to confirm the presence of autofluorescent cells. Initial analysis showed that 14.7% of the splenocytes exhibited autofluorescence, a known feature of RPMs. In contrast, peripheral blood mononuclear cells (PBMCs) showed no autofluorescence (0%), indicating this characteristic is specific to splenic RPMs (**Figure 1**).

To isolate RPMs from pig spleen tissue, we employed two distinct methods: (1) CD163 antibody-based sorting, and (2) magnetic-activated cell sorting (MACS) leveraging iron-induced magnetic properties. Although autofluorescence was used to validate RPM identity, it was not used for sorting in the MACS-based method.

**Figure 2:**
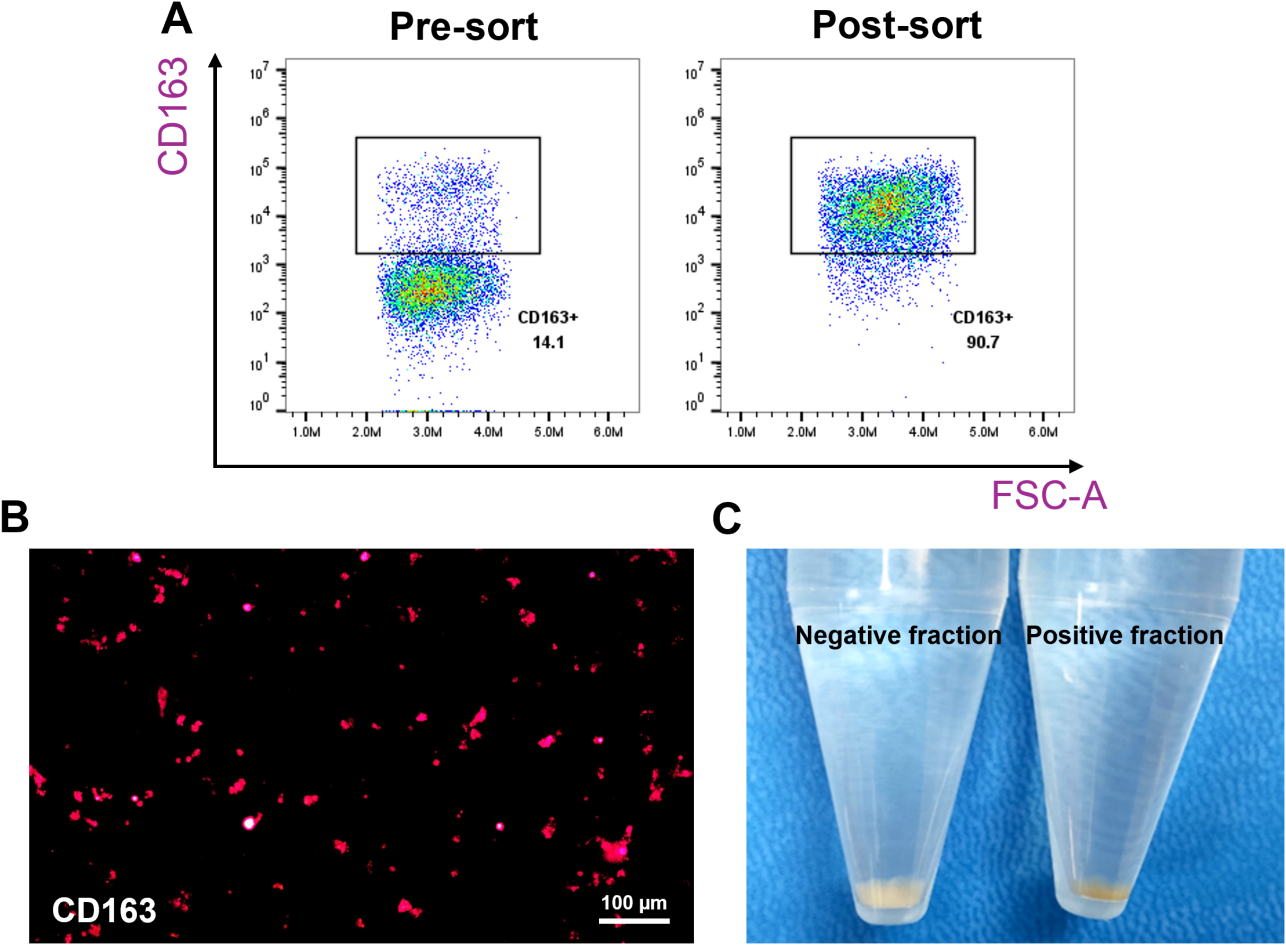
Enrichment of CD163^+^ macrophages from pig spleen. Splenocytes were incubated with purified anti-pig CD163 antibodies, followed by labeling with Ig-conjugated microbeads, and passed through a MACS column. Positive fractions (CD163-enriched) and negative fractions were collected for comparison. The expression of CD163 was evaluated using flow cytometry and fluorescence microscopy. **(A)** Representative flow cytometry plots showing the percentage of CD163^+^ cells before and after enrichment. **(B)** Fluorescence microscopy image (200X magnification) confirming CD163 expression in the positive fraction. **(C)** Images showing the color difference between the positive and negative fractions. n = 3 independent experiments using cells from three individual pigs.

**Figure 3:**
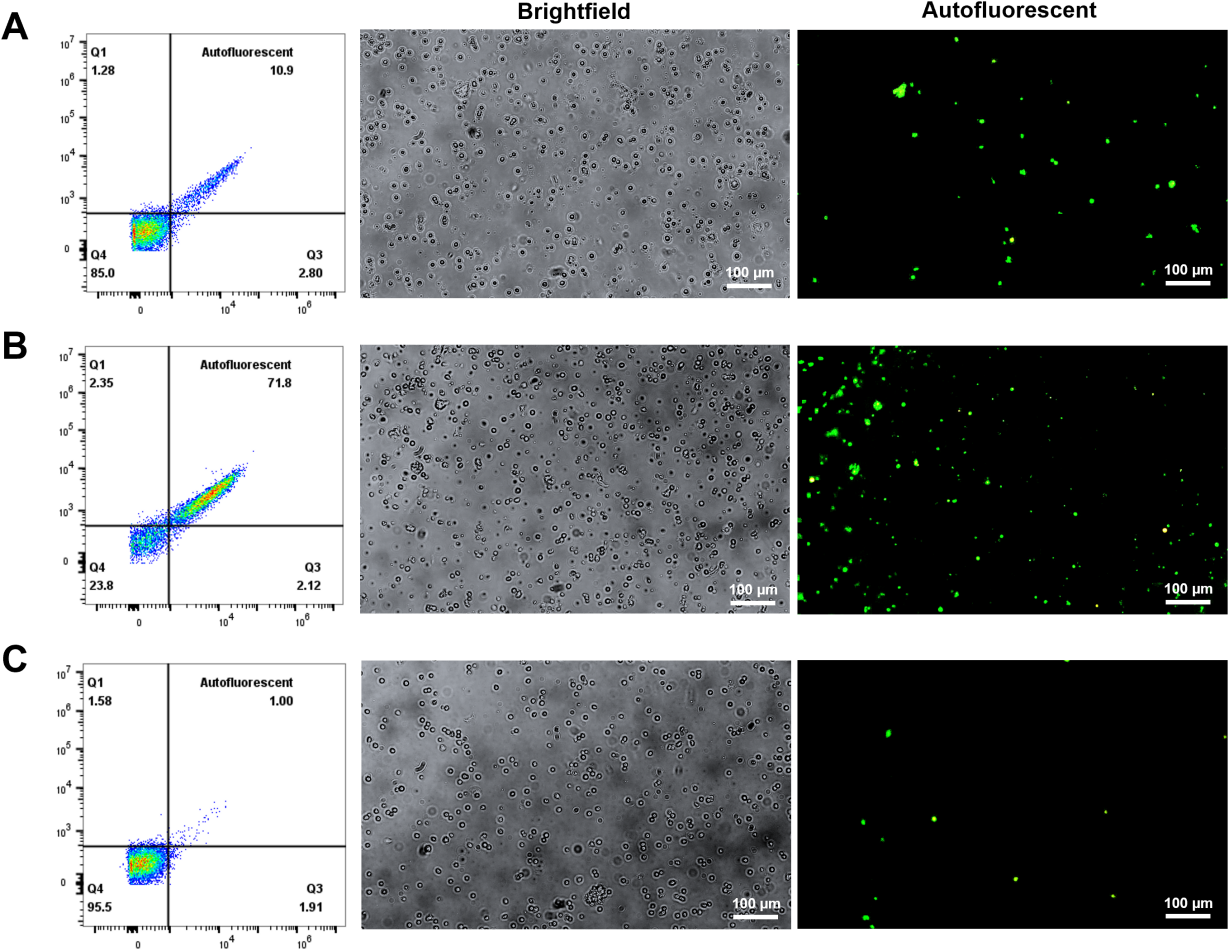
Autofluorescent cell population in different fractions after CD163 enrichment. Splenocytes were incubated with purified anti-pig CD163 antibodies and Ig-conjugated microbeads, then sorted using a MACS column to collect both positive and negative fractions. The cells were subsequently cultured in RPMI 1640 medium. Autofluorescence was assessed in unstained cells using a fluorescence microscope (200X magnification) to identify autofluorescent RPMs. **(A)** Pre-sort fraction; **(B)** Post-sort positive fraction; **(C)** Post-sort negative fraction. n = 3 independent experiments using cells from three individual pigs.

The CD163-based method involved labelling splenocytes with anti-CD163 antibodies and magnetic beads, followed by MACS enrichment. CD163^+^ cells comprised ~14.1% of pre-sort cells, which increased to ~90% post-sorting (**Figures 2A, B**). The CD163^+^ fraction appeared rusty brown, consistent with intracellular iron accumulation (**Figure 2C**) (17, 18). Of these sorted cells, 71.8% were autofluorescent (**Figure 3**).

**Figure 4:**
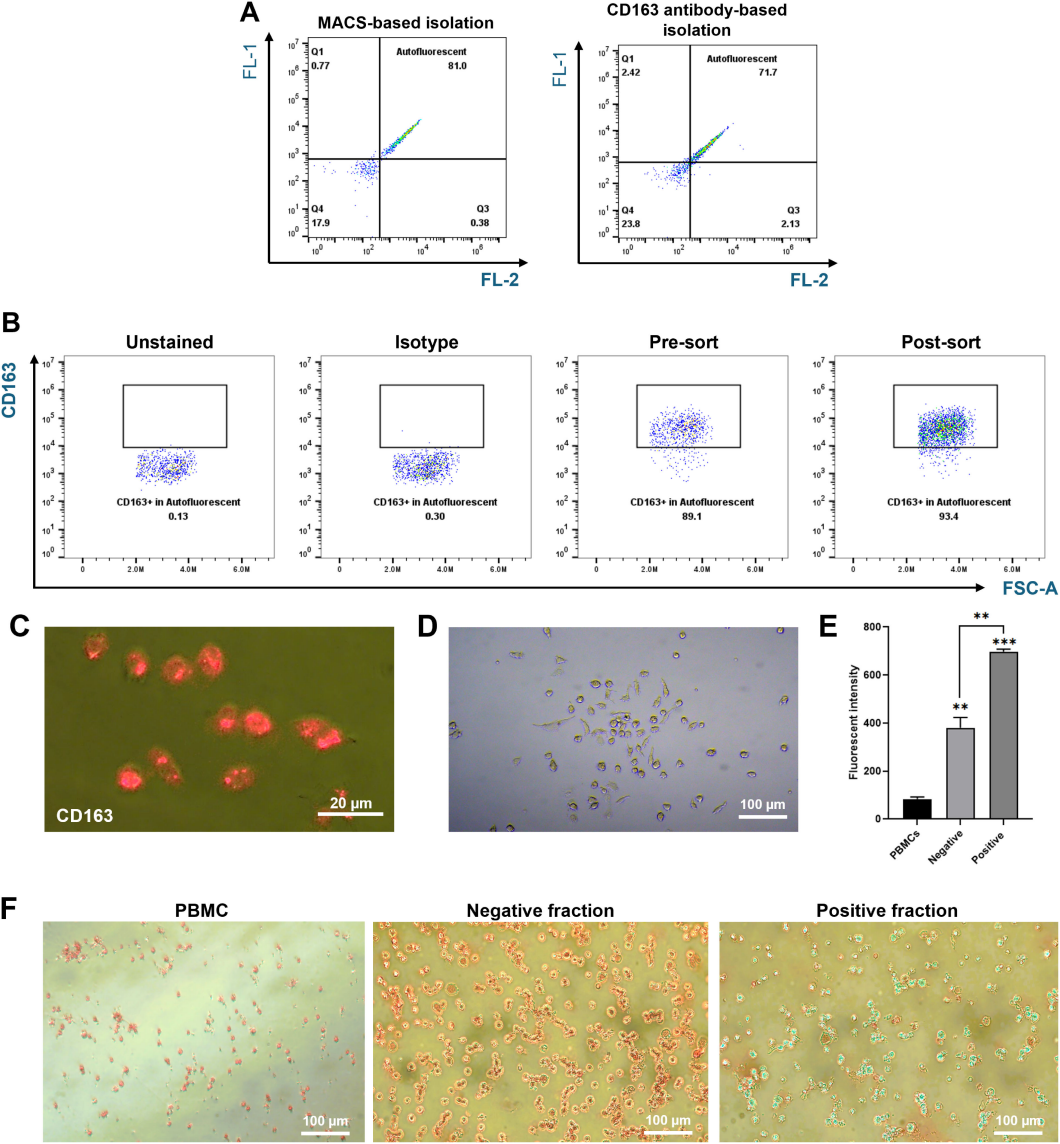
Characterization of autofluorescent cells isolated using MACS. Splenocytes were passed through a MACS column, and the positive fraction was collected. **(A)** Comparison of autofluorescent cell populations between the MACS-positive fraction (left) and the CD163-positive fraction (right). **(B)** Representative flow cytometry plots showing the percentage of CD163-positive cells within the autofluorescent population. The positive fraction was stained with PE-conjugated anti-pig CD163 antibodies, and CD163 expression levels were measured by flow cytometry. **(C)** CD163 expression levels in autofluorescent cells were further assessed after culturing the sorted cells for 3 days and staining with PE-conjugated CD163 antibodies. Images were captured using a fluorescence microscope at 400X magnification. **(D)** Cell morphology of cultured autofluorescent cells was observed using a fluorescence microscope at 200X magnification. **(E, F)** Ferric iron content in the positive fraction compared to the negative fraction was measured using the AUR assay **(E)** and visualized through Prussian Blue staining **(F)**. Images were captured at 200X magnification. **p < 0.01, ***p < 0.001. n = 3 independent experiments using cells from three individual pigs.

The alternative magnetic isolation approach was designed to exploit RPMs’ high iron content, which confers magnetic properties, allowing for their retention in the MACS column without antibodies. This method captured cells with high autofluorescence (81%) and typical macrophage morphology. Prussian blue staining validated the presence of ferric iron (**Figure 4**). Thus, this MACS-based approach—driven by magnetic properties and validated by autofluorescence—provides a cost-effective and efficient strategy for isolating RPMs.

### 3.2 Marker characterization of isolated RPMs

**Figure 5:**
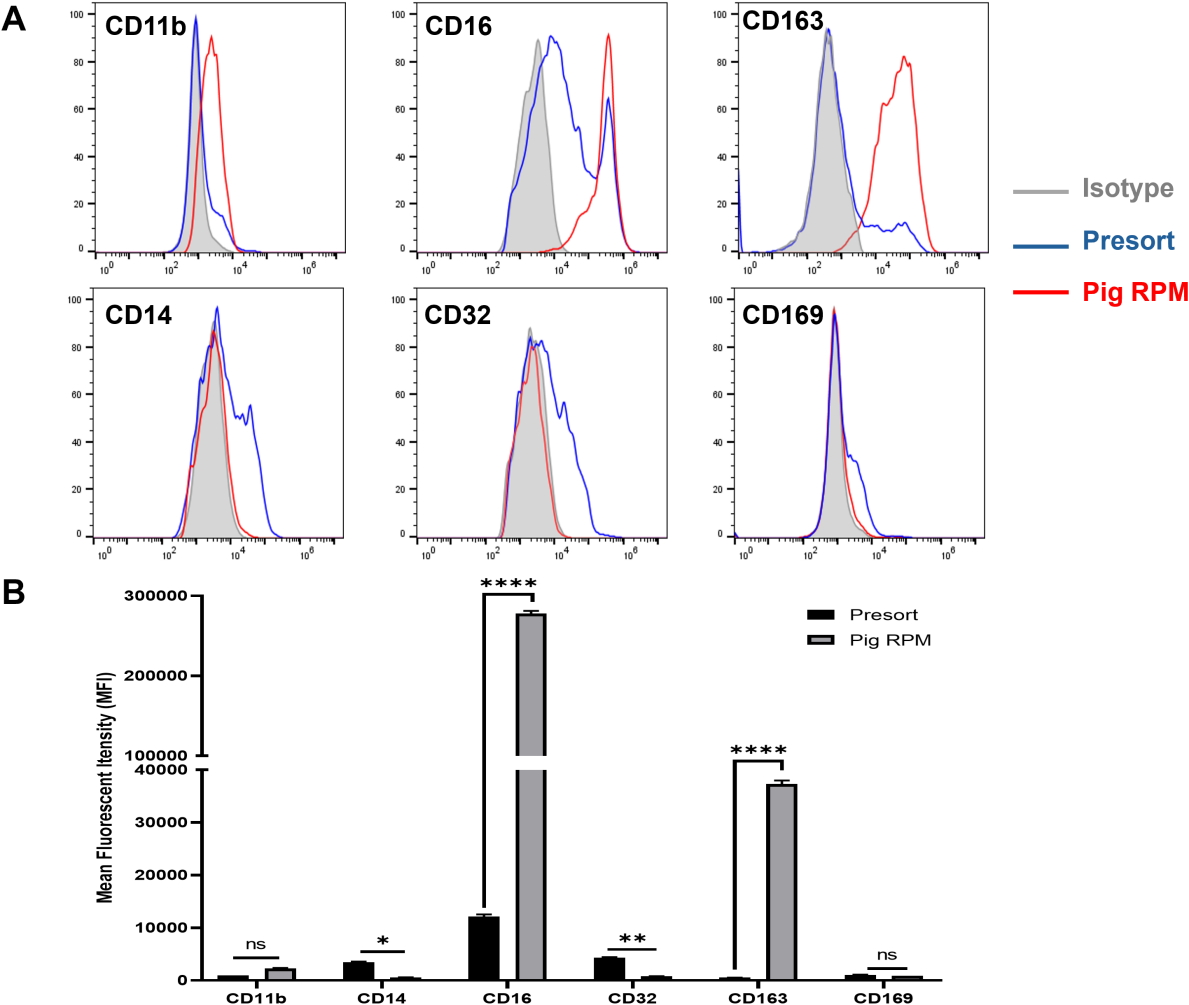
Phenotypic characterization of pig RPMs. Splenocytes were passed through a MACS column, and the positive fraction was collected. Both pre-sort and post-sort cells were subsequently stained with antibodies targeting CD11b, CD14, CD16, CD32, CD163, and CD169 to evaluate surface marker expression. Flow cytometry was performed to assess the phenotypic profile of the isolated RPMs. **(A)** Representative histograms: red line for post-sort (RPMs), blue for pre-sort (splenocytes), and gray for isotype controls; **(B)** MFI expression of markers on splenocyte (presort) and pig RPM. *p< 0.05; **p<0.01; ***p<0.001 ****p< 0.0001 (n=3). n = 3 independent experiments using cells from three individual pigs.

We further characterized the marker expression in isolated autofluorescent cells using the MACS-based method. In humans, RPMs are characterized by low levels of CD14, CD32, CD169, moderate or low expression of CD11b, and high expression of CD16 and CD163 (3, 12). In line with this, we assessed these markers on pig splenic autofluorescent cells. Our results indicated that pig RPMs exhibit high expression of CD163 and CD16, moderate levels of CD11b, and very low or undetectable levels of CD14, CD32, and CD169 (**Figure 5**). These findings suggest a conserved marker profile between pig and human RPMs, supporting the fidelity of our isolation method.

### 3.3 Functional validation of RPMs through erythrophagocytosis

**Figure 6:**
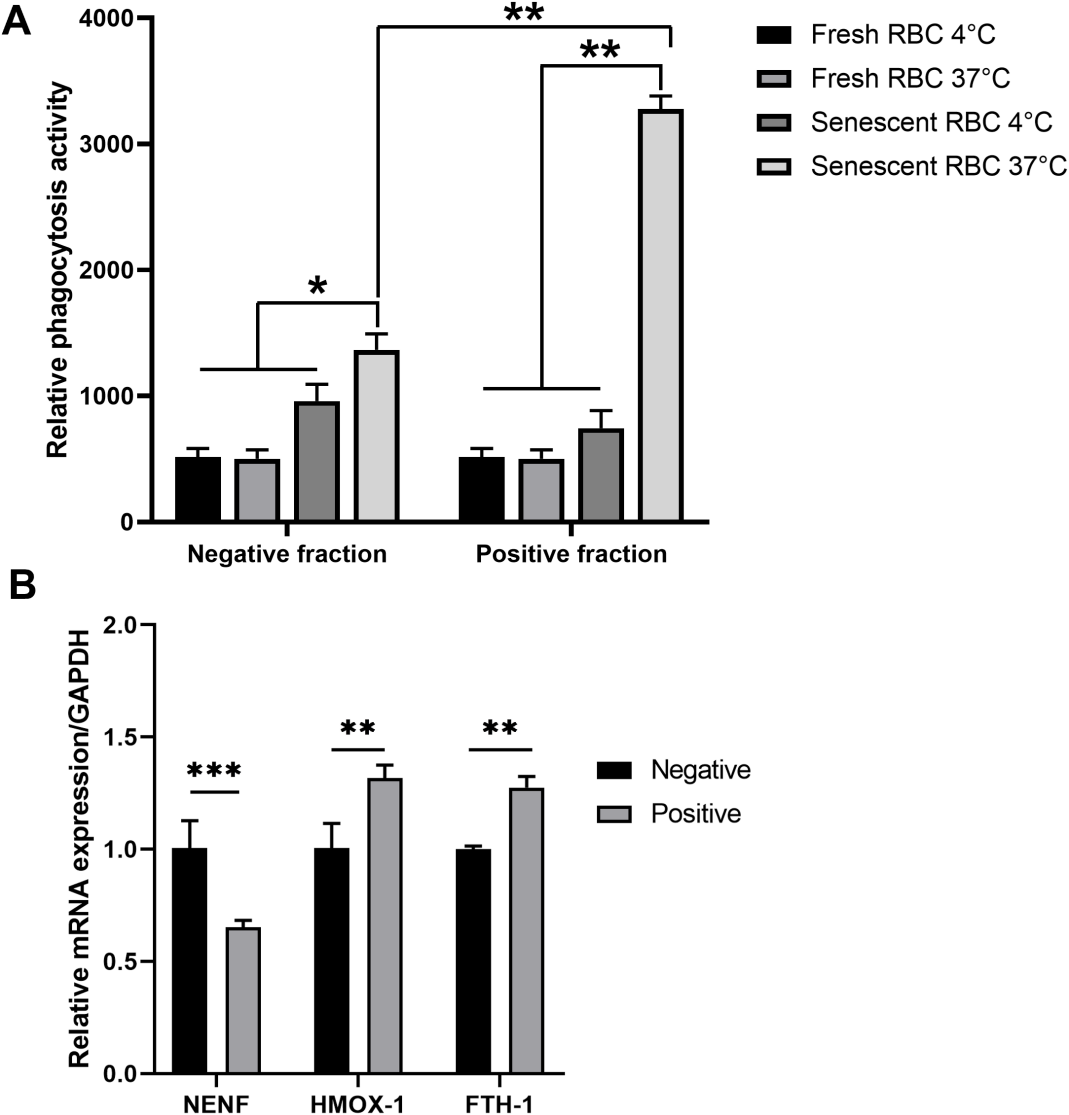
Phagocytic activity of the autofluorescence-positive fraction against RBCs. Following MACS isolation, both positive and negative fractions of splenocytes were collected. **(A)** The sorted cells were co-cultured with calcein AM-labeled freshly isolated RBCs (fresh RBCs) or six-month-old RBCs (senescent RBCs) from triple knockout (TKO) pigs. After 1 hour, the fluorescent signal from phagocytosed RBCs was measured using a microplate reader. **(B)** RNA was extracted from collected cells, and the expression levels of *NENF*, *HMOX-1*, and *FTH1* were assessed by real-time PCR. *p < 0.05; **p < 0.01; ***p < 0.001. n = 3 independent experiments using cells from three individual pigs.

To confirm the functional role of RPMs in clearing senescent red blood cells (RBCs), we evaluated their phagocytic activity using six-month-stored RBCs as a model for senescent RBCs and freshly prepared RBCs as a control. Phagocytosis assays were performed at 37°C to facilitate phagocytosis and at 4°C as a negative control to inhibit the process. The results demonstrated that RPMs displayed a significantly higher phagocytic capacity against senescent RBCs compared to the non-autofluorescent negative population, while no phagocytosis was observed with freshly prepared RBCs (**Figure 6A**). This suggests that autofluorescent RPMs are actively involved in RBC clearance, a key functional characteristic of these cells.

We further analyzed the expression of genes related to iron recycling and immune responses. The autofluorescent RPM population showed upregulated expressions of *HMOX-1*, encoding heme oxygenase-1 (17), and *FTH1*, encoding ferritin heavy chain (19), both essential for heme and iron metabolism. Conversely, *NENF*, a gene encoding neudesin and known to be suppressed in RPMs (3), was significantly downregulated in the autofluorescent-positive fraction compared to non-RPMs (**Figure 6B**). These gene expression patterns are consistent with the functional roles of RPMs, supporting their involvement in iron processing and their unique genetic signature within the spleen.

## 4 Discussion

In this study, we developed and compared two methods for isolating red pulp macrophages (RPMs) from pig spleen: CD163 antibody-based magnetic separation and magnetic property-based MACS enrichment. While CD163-based sorting effectively enriched RPMs, a significant proportion of the CD163^+^ population lacked autofluorescence, indicating possible heterogeneity or inclusion of non-RPM subsets.

In contrast, the MACS-based approach isolated RPMs based on their intrinsic magnetic properties derived from intracellular iron accumulation following erythrophagocytosis. Autofluorescence was not used as a sorting criterion but served as a reliable validation marker post-isolation. This distinction is important: the magnetic enrichment was based on functional iron content, not optical fluorescence. While other splenic cell types may transiently phagocytose RBCs (20), they lack the sustained iron accumulation and autofluorescence characteristic of bona fide RPMs (21, 22).

The MACS-based method yielded a higher proportion of autofluorescent RPMs (81%) than the CD163-based method (71.8%), and 93% of MACS-enriched autofluorescent cells expressed CD163. This suggests some RPM heterogeneity or the possible presence of additional macrophage subsets with iron retention and autofluorescence. To further enhance enrichment, several protocol refinements could be considered. For example, adjusting magnetic column parameters—including flow rate, magnet strength, and the use of sequential columns—may improve cell capture. In addition, incorporating pre-enrichment steps to deplete non-macrophage splenocytes before magnetic sorting could enhance the purity of the isolated population. These adjustments could help increase both yield and specificity, while maintaining the simplicity, scalability, and cost-effectiveness of the MACS-based method.

The CD163-based method remains valuable for isolating specific RPM subtypes; however, it is more time-consuming, reagent-intensive, and expensive. The MACS-based approach, by contrast, offers simplicity, scalability, and reduced cost—making it especially suitable for high-throughput or large-scale studies.

By establishing a magnetic property-based RPM isolation protocol validated by autofluorescence, our study aligns with and extends murine findings (13) to a large-animal model. These findings support the translational utility of pig RPMs for studying iron metabolism and immune responses. Future work using single-cell approaches will further resolve the cellular heterogeneity within the RPM population and may aid in refining isolation strategies.

The marker profile of isolated pig RPMs in our study shares several similarities with human RPMs, which are also described as CD163^hi^, CD16^hi^, and CD14^lo^ or negative (3, 12). In human spleen tissue, RPMs are further distinguished by low levels of CD32 and CD169, aligning with our findings in pigs. In murine models, RPMs typically express F4/80^hi^, CD11b^low^ to intermediate, and lack CD14 and CD169 expression as well (13, 17). Therefore, the marker profile of pig RPMs reported here suggests a conserved immunophenotype across species, supporting the relevance of the pig model in RPM-related immunological and hematological research.

Beyond methodological optimization, the magnetic property-based approach offers several advantages with broad applicability. First, it enables the isolation of functionally defined macrophage populations without relying on species-specific antibodies, which are often unavailable or costly—particularly for large or non-model animals. Second, given the central role of iron-handling macrophages in diverse physiological and pathological contexts (e.g., anemia, inflammation, iron overload disorders, aging, and infection), this method facilitates the direct *ex vivo* isolation of functionally active cells for downstream applications such as transcriptomics, functional assays, and comparative immunology. This, in turn, can help align preclinical animal models more closely with human biology. Furthermore, the ability to isolate RPMs from large animals such as pigs—whose immunological and hematological parameters closely resemble those of humans—enhances the translational value of these models and may help bridge the gap between preclinical and clinical research.

**Figure 7:**
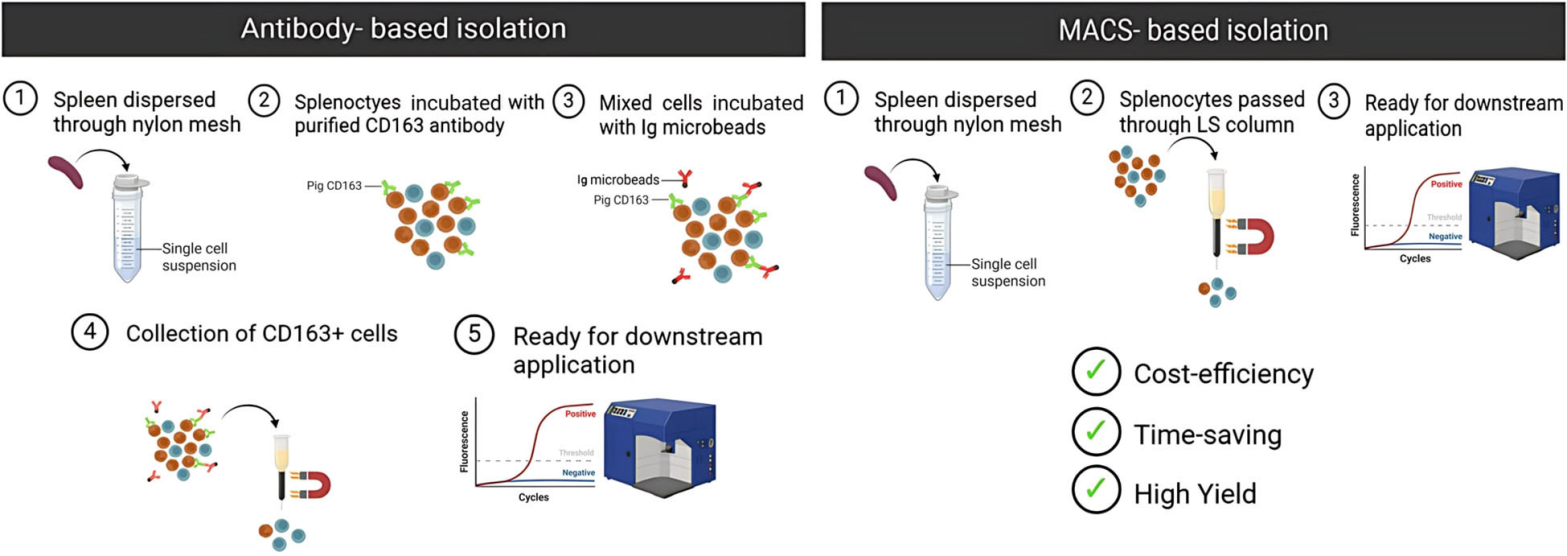
Comparison of antibody-based and MACS-based isolation methods for pig RPMs. The MACS-based method offers advantages over the antibody-based approach, including cost-efficiency, time-saving, and higher yield, making it more suitable for large-scale studies.

In summary, this study demonstrates that magnetic property-based enrichment, confirmed by autofluorescence and functional assays, provides a robust, economical, and scalable method for isolating functionally active RPMs from pig spleen (**Figure 7**). This strategy offers a valuable tool for comparative immunology and translational research involving tissue-resident macrophages.

## Data Availability

The original contributions presented in the study are included in the article/**Supplementary Material**. Further inquiries can be directed to the corresponding authors.
