# Supplementary material for: Comparative evaluation of isolation techniques and characterization of red pulp macrophages from pig splenocytes
Source: Front Immunol. 2025 Sep 1;16:1617203. doi: 10.3389/fimmu.2025.1617203 (PMC12434077; doi:10.3389/fimmu.2025.1617203)
Supplement: Supplementary file 1 [file DataSheet1.pdf]

frontiers

# *Supplementary Material*

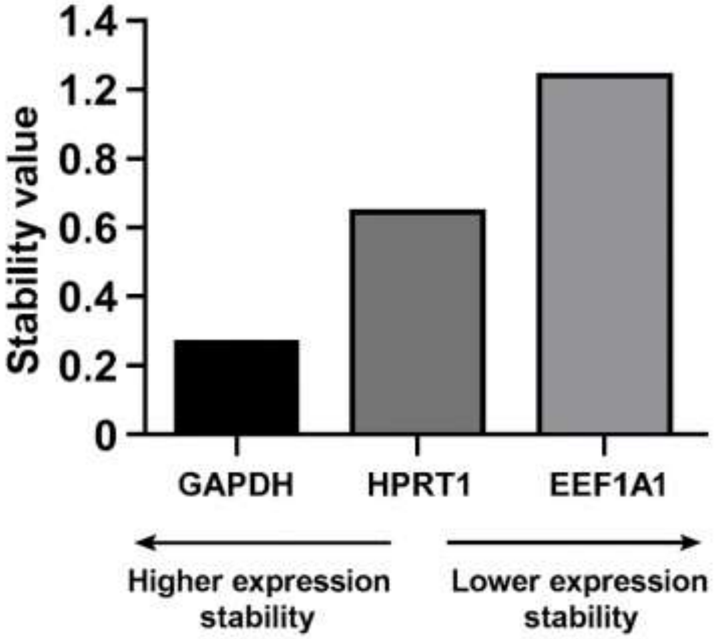


**Supplementary Figure 1: Expression stability of reference genes *GAPDH*, *HPRT1*, and *EEF1A1* in pig splenic tissue**. Gene expression levels were quantified by real-time RT-PCR, and expression stability was analyzed using NormFinder algorithms. Lower stability values indicate more stable expression.
